# Supplementary material for: Gut Microbiome of Healthy and Arthritic Dogs
Source: Vet Sci. 2020 Jul 14;7(3):92. doi: 10.3390/vetsci7030092 (PMC7558702; doi:10.3390/vetsci7030092)
Supplement: Supplementary file 1 [file vetsci-07-00092-s001.pdf]

Supplementary Table S1. Health status, breed, sex, weight and age of the dogs recruited for the study. Average live weight was 29.2±8.2 kg and 20.1±7.6 kg for AD and HD group, respectively. Means are significantly different for $p<0.05$.

Status	Breed	Sex	Live weight, kg	Age, years
AD	Drahtaar	FS	28.0	9
AD	Labrador Retriever	FS	20.0	6
AD	Mixed Breed	FS	30.0	7
AD	Dobermann	FS	34.0	8
AD	Hovawart	MC	33.0	11
AD	Labrador Retriever	MC	38.0	10
AD	German Sheperd	FS	25.0	13
AD	Labrador Retriever	MC	36.0	2
AD	Border Collie	FS	21.0	6
AD	Mixed Breed	MC	28.5	4
AD	Labrador Retriever	MC	42.0	6
AD	Pitbull Terrier	FS	31.0	10
AD	Crossbreed	MC	10.0	4
AD	Golden Retriever	FS	33.0	4
HD	Border Collie	FS	20.0	10
HD	Border Collie	FS	20.0	7
HD	Golden Retriever	FS	25.0	10
HD	German Sheperd	FS	25.0	2
HD	Golden Retriever	FS	30.0	10
HD	Golden Retriever	FS	30.0	3
HD	Golden Retriever	FS	25.0	2
HD	Cocker Spaniel	M	14.0	10
HD	Border Collie	F	10.0	13
HD	Mixed Breed	F	11.0	2
HD	Mixed Breed	F	12.8	4
HD	Border Collie	F	10.9	6
HD	German Shepherd	M	28.0	8

AD: dogs with osteoarthritis; HD: healthy dogs.

Supplementary Table S2. Mean concentrations (µmol/g) and molar proportions (%) of lactate and short chain fatty acids in the feces of the dogs sampled at the beginning of the study (T0) and after 45 (T45) days.

Item	Unit	T0		T45		[1]SEM	Effects		
		AD	HD	AD	HD		Time	Status	Time X Status
Lactate	µmol/g	1.7	2.6	10.9	1.7	1.67	NS	NS	NS
Acetate		148.5	135.9	145.2	114.3	11.90	NS	NS	NS
Propionate		56.7	50.0	61.0	55.4	4.67	NS	NS	NS
Isobutyrate		48.2	28.9	40.4	40.1	6.33	NS	NS	NS
Butyrate		18.6	12.9	20.9	13.2	1.34	NS	NS	NS
Isovalerate		4.1	5.8	9.4	7.2	0.96	NS	NS	NS
Total		277.8	236.0	287.8	231.9	21.43	NS	NS	NS
Lactate	%	0.6	1.0	5.4	1.2	0.95	NS	NS	NS
Acetate		53.7	55.1	49.3	48.3	1.49	*	NS	NS
Propionate		21.3	21.2	21.8	25.6	0.88	NS	NS	NS
Isobutyrate		15.4	13.1	11.8	13.9	1.34	NS	NS	NS
Butyrate		7.4	6.6	8.0	7.4	0.55	NS	*	NS
Isovalerate		1.5	3.1	3.8	3.7	0.44	NS	NS	NS

[1] SEM: standard error of the means. p <0.05; **: p < 0.001; NS: Not Significant.

AD: dogs with osteoarthritis; HD: healthy dogs

Supplementary Table S3. Relative abundances of the prevalent families detected in dog feces samples at the beginning of the study (T0) and at the end (T45).

Family	T0		T45	
	AD	HD	AD	HD
Unknown	4.4 [a]	5.4 [a]	4.7 [a]	8.9 [a]
Alcaligenaceae	17.9 [a]	14.0 [a]	20.6 [a]	19.9 [a]
Anaeroplasmataceae	0.4 [a]	0.2 [a]	0.4 [a]	1.9 [b]
Bacteroidaceae	21.3 [ab]	14.1 [a]	22.5 [ab]	31.7 [b]
Clostridiaceae	36.6 [ab]	32.0 [b]	27.5 [ab]	23.2 [a]
Coriobacteriaceae	3.2 [b]	2.4 [ab]	3.2 [b]	1.5 [a]
Enterobacteriaceae	3.0 [ab]	1.0 [a]	8.8 [b]	1.9 [ab]
Enterococcaceae	1.4 [a]	0.7 [a]	1.0 [ab]	9.5 [b]
Erysipelotrichaceae	4.7 [b]	9.6 [a]	5.1 [b]	4.8 [b]
f_Bacteria	0.3 [a]	2.5 [b]	0.5 [a]	5.6 [b]
Fusobacteriaceae	35.9 [ab]	26.2 [a]	40.5 [b]	30.3 [ab]
Helicobacteraceae	1.4 [a]	0.6 [a]	2.3 [a]	0.4 [a]
Lachnospiraceae	9.8 [b]	12.9 [b]	10.9 [ab]	9.5 [b]
Lactobacillaceae	6.4 [a]	3.4 [a]	1.1 [a]	5.0 [a]
Mogibacteriaceae	2.2 [a]	16.2 [b]	4.0 [a]	11.9 [b]
Odoribacteraceae	0.2 [a]	0.5 [ab]	0.7 [a]	1.9 [b]
Paraprevotellaceae	10.2 [a]	20.1 [b]	10.8 [a]	18.7 [b]

Peptococcaceae	0.6	a	7.9	b	2.1	a	6.3	b
Peptostreptococcaceae	17.6	a	36.7	a	29.6	a	21.8	a
Porphyromonadaceae	2.5	a	6.9	b	1.7	a	7.7	b
Prevotellaceae	43.7	a	33.0	a	37.6	a	38.5	a
Ruminococcaceae	6.4	a	8.4	ab	7.6	a	11.4	b
S24-7	2.0	a	8.7	b	2.8	ab	5.2	b
Streptococcaceae	20.7	ab	0.4	b	22.2	b	6.4	b
Succinivibrionaceae	1.2	a	4.0	b	0.8	a	2.1	ab
Turicibacteraceae	8.5	a	26.3	b	11.5	a	5.9	a
Veillonellaceae	36.8	b	14.3	a	34.1	b	19.0	ab

AD: dogs with osteoarthritis; HD: healthy dogs

Supplementary Table S4. Relative abundances of the prevalent genera detected in dog feces samples at the beginning of the study (T0) and at the end (T45).

Genus	T0				T45			
	AD		HD		AD		HD	
Adlercreutzia	0.5	a	0.8	a	0.4	a	0.2	a
Allobaculum	11.1	a	44.7	a	9.5	a	8.4	a
Anaerobiospirillum	0.7	a	11.0	b	0.3	a	5.5	b
Anaerofilum	0.7	a	0.6	a	0.6	a	0.5	a
Bacteroides	23.4	ab	15.9	a	24.4	ab	36.8	b
Blautia	18.5	a	31.0	b	21.3	ab	22.5	ab
Butyricicoccus	1.2	a	0.8	a	0.7	a	1.0	a
Catenibacterium	6.7	a	7.3	a	14.4	a	8.9	a
Clostridium	70.5	a	49.7	a	49.5	a	41.5	a
Collinsella	6.7	a	3.6	a	6.7	a	2.9	a
Coprobacillus	2.0	a	1.4	a	1.7	a	1.6	a
Coprococcus	3.8	a	2.8	a	4.4	a	6.1	a
Dorea	27.2	a	25.6	a	27.8	a	15.2	a
Enterococcus	1.4	a	0.7	a	1.0	a	9.5	a
Epulopiscium	0.1	a	0.9	a	0.5	a	1.0	a
Escherichia	3.0	ab	1.0	a	8.8	b	1.9	ab
Eubacterium	2.1	a	4.4	a	3.1	a	4.4	a
Faecalibacterium	24.8	a	20.7	a	32.1	a	33.8	a
Fusobacterium	107.5	a	78.3	a	121.2	a	90.9	a
Helicobacter	1.4	a	0.6	a	2.3	a	0.4	a
Lachnospira	2.6	ab	1.6	ab	5.3	b	1.1	a
Lactobacillus	6.4	a	3.4	a	1.1	a	5.0	a
Megamonas	65.6	ab	18.2	a	61.5	b	19.1	ab
Odoribacter	0.2	a	0.5	ab	0.7	ab	1.9	b

Oscillospira	2.2 [a]	4.1 [ab]	2.2 [ab]	8.9 [b]
p-75-a5	1.4 [ab]	4.9 [b]	1.5 [a]	4.0 [ab]
Parabacteroides	2.5 [a]	6.9 [ab]	1.7 [a]	7.7 [b]
Paraprevotella	0.1 [a]	0.7 [a]	0.4 [a]	0.7 [a]
Peptococcus	0.6 [a]	7.9 [c]	2.1 [ab]	6.3 [bc]
Phascolarctobacterium	8.0 [a]	10.3 [ab]	6.7 [a]	18.9 [b]
Prevotella	36.0 [a]	37.1 [a]	33.7 [a]	39.6 [a]
Roseburia	1.0 [a]	0.3 [a]	0.6 [a]	0.5 [a]
Ruminococcus	4.4 [a]	5.4 [a]	4.9 [a]	3.6 [a]
Sarcina	2.9 [a]	0.2 [a]	3.5 [a]	0.3 [a]
Slackia	1.7 [a]	2.5 [a]	1.9 [a]	1.6 [a]
SMB53	1.6 [a]	4.8 [a]	3.0 [a]	3.5 [a]
Streptococcus	20.7 [ab]	0.4 [a]	22.2 [b]	6.4 [ab]
Sutterella	17.9 [a]	14.0 [a]	20.6 [a]	19.9 [a]
Turicibacter	8.5 [a]	26.3 [b]	11.5 [a]	5.9 [ab]

AD: dogs with osteoarthritis; HD: healthy dogs
